# Discovery of anti-inflammatory agents from *Oreorchis patens*, a medicinal and edible plant: mechanistic insights and potential therapeutic applications

**DOI:** 10.3389/fnut.2026.1781206

**Published:** 2026-03-23

**Authors:** Fan Wei, Ruonan Wang, Yiming Liu, Hui Ren, Wenwen Yin, Hongyu Sun, Wenyu Zhao, Shijun Li

**Affiliations:** 1 Department of Clinical Laboratory, The First Affiliated Hospital of Dalian Medical University, Dalian, China; 2 College (Institute) of Integrative Medicine, Dalian Medical University, Dalian, China; 3 School of Traditional Chinese Materia Medica, Shenyang Pharmaceutical University, Shenyang, China

**Keywords:** AMPK, bioactive compounds, food and medicine homology, inflammation, *Oreorchis patens*, phenanthrene derivatives

## Abstract

Bioactive dietary compounds serve as beneficial supplements for managing chronic inflammation, and plants with both edible and medicinal properties represent a safe and effective source of these compounds. In this study, metabolomic investigation of the edible parts of *Oreorchis patens*, a plant classified in China as having food and medicine homology, led to the identification of six phenanthrene derivatives, including two monomers and four dimers. Their planar structures and absolute configurations were established by nuclear magnetic resonance spectroscopy and electronic circular dichroism, supported by computational chemistry analyses. Among these compounds, phenanthrene dimer **3** exhibited notable anti-inflammatory activity in lipopolysaccharide-stimulated murine alveolar (MH-S) macrophages. More importantly, compound **3** was found to stably bind to the allosteric drug and metabolite site of adenosine monophosphate-activated protein kinase (AMPK), thereby protecting the kinase from dephosphorylation and promoting its activation. Activated AMPK was subsequently shown to regulate NF-κB signaling, resulting in pronounced anti-inflammatory effects. Collectively, these findings reveal nutritionally valuable bioactive compounds from a food and medicine homologous plant that act as AMPK modulators with anti-inflammatory potential.

## 1 Introduction

*Oreorchis patens* (Lindl.) Lindl., a member of the Orchidaceae family, is distributed throughout East Asia, and its pseudobulbs have long been consumed as a food source in China, Korea, and Japan due to their high starch content. Beyond serving as a source of calories, the pseudobulbs of *O. patens* possess high nutritional value, as their secondary metabolites—including steroids, flavonoids, and fatty acid derivatives—exhibit notable antioxidant and anti-inflammatory activities (1). Therefore, *O. patens* is recognized as a food and medicine homology for its edible and medicinal properties, and is recommended as a functional food supplement for individuals with pneumonia and cancer (2). Systematic characterization of the phytochemistry of *O. patens* pseudobulbs could therefore enhance their economic value while informing sustainable industrial applications.

Controlling inflammation is a treatment approach for numerous conditions, including neurodegenerative and autoimmune diseases, such as Parkinson’s disease and rheumatoid arthritis (3). Typical anti-inflammatory medications, including corticosteroids and non-steroidal anti-inflammatory drugs (NSAIDs), have well-documented side effects. As a result, scientists are investigating the anti-inflammatory properties of natural products from food sources. For example, *Cistanche deserticola* Ma, a food and medicine homology plant used in northern China, has been reported to attenuate dextran sulfate sodium (DSS)–induced inflammatory bowel disease through its total glycosides (4). Polysaccharides from *Platycodon grandiflorum* (Jacq.) A. de Candolle exert hepatoprotective effects by modulating anti-inflammatory responses and enhancing antioxidant activity (5). Natural flavonoids from food sources, and saponins from *Nymphoides hydrophyllum* (Lour.) Kuntze, an edible aquatic plant in Taiwan, possess anti-inflammatory potential through elastase inhibition (6, 7). Finally, caffeic acid, a natural product that is widely found in food and beverages, alleviates lung inflammation and fibrosis by targeting the annexin A5 protein (8).

Adenosine monophosphate-activated protein kinase (AMPK), a Ser/Thr protein kinase, functions to monitor cellular energy and regulates metabolism. AMPK becomes activated when cellular energy levels are low, initiating pathways that boost energy production and suppress energy-consuming processes (9, 10). The activated form of AMPK, known as phosphorylated AMPK, can regulate metabolism by phosphorylating the downstream acetyl-CoA carboxylase (ACC), which increases glucose and fatty acid absorption, boosts glycolysis and fatty acid oxidation, and suppresses glycogen and protein synthesis (11). During inflammation, AMPK activation inhibits the c-Jun N-terminal kinase (JNK) and nuclear factor kappa-light-chain-enhancer of activated B cells (NF-κB) signaling cascade, leading to decreased mRNA and protein levels of pro-inflammatory cytokines, including tumor necrosis factor alpha (TNF-*α*) and interleukin (IL)-6. This inhibition helps to alleviate excessive inflammatory responses in conditions such as cardiac ischemia/reperfusion and chronic pain (12, 13). Therefore, AMPK is considered a potential target for inflammatory diseases, including hepatitis and enteritis (14, 15).

**Figure 1:**
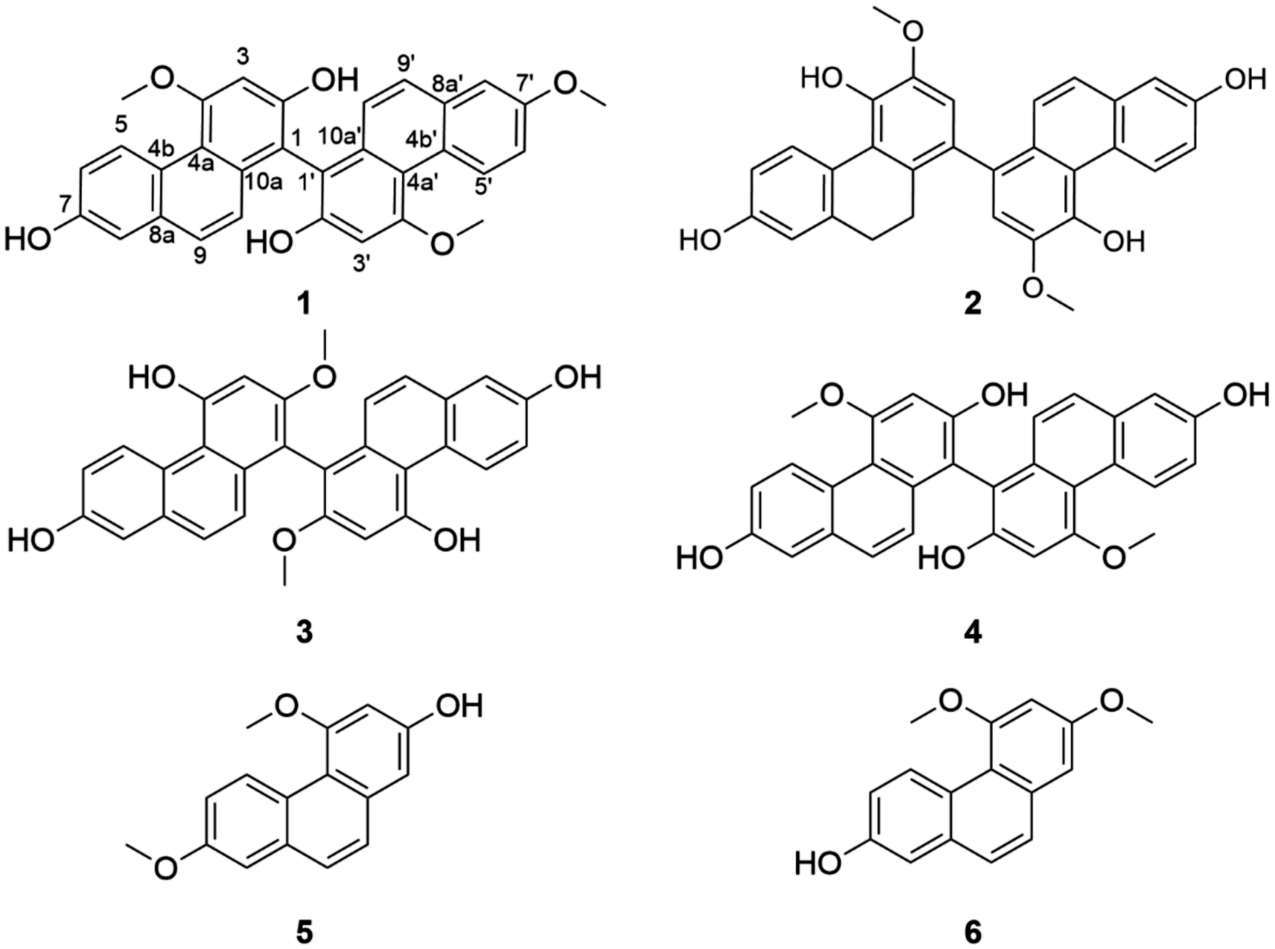
The secondary metabolites obtained from the pseudobulbs of *O. patens*.

Although *O. patens* has a long-standing history as a food and medicine homology in traditional folk practice, scientific reports on its bioactivity remain scarce due to its restricted geographical distribution. Currently, the functional potential of *O. patens* is largely supported by empirical folk knowledge rather than molecular evidence. A study has indicated that *O. patens* extracts possess therapeutic effects against non-alcoholic fatty liver disease (NAFLD) in murine models, significantly reducing hepatic inflammatory cell infiltration (16). Furthermore, polysaccharides of *O. patens* have been shown to restore immune function in cyclophosphamide-induced immunosuppressed mice (17). However, these preliminary studies focus primarily on crude extracts, leaving a significant gap in the identification of specific small-molecule active constituents and the elucidation of their underlying molecular mechanisms. In this study, a systematic chemical exploration of edible parts of *O. patens* was performed, yielding six phenanthrene derivatives, including two monomers (compounds **5** and **6**) (Figure 1) and four dimers (compounds **1**–**4**). All six compounds were isolated from *O. patens* for the first time. Dimers **1**–**4** were found to be atropisomeric mixtures and were successfully separated by chiral chromatography. Three pairs, compounds **1a/1b**, **2a/2b**, and **4a/4b**, possessed unreported absolute configurations, which were established by electron circular dichroism (ECD) assisted by computational chemistry. More importantly, phenanthrene dimer **3** significantly reduced the secretion of inflammatory factors, demonstrating a strong anti-inflammatory effect in lipopolysaccharide (LPS)-stimulated murine alveolar (MH-S) macrophages. Initial studies show that compound **3** binds stably to the AMPK protein, activating it and suppressing NF-κB signaling, resulting in anti-inflammatory effects. This report presents a new example of functional anti-inflammatory components derived from food and medicine homology.

## 2 Materials and methods

### 2.1 Reagents

Methanol, dichloromethane, acetonitrile, *n*-hexane, ethanol, and DMSO were purchased from Energy Chemicals (Anhui, China). Silica gel (100–200 mesh) was obtained from Bangkai Chemical Industrials, China. Preparative high-performance liquid chromatography (HPLC) was performed using an SP-5030 instrument equipped with a dual-pump system and a UV detector (Separation Technology Co., Beijing, China). Reversed-phase HPLC was performed using a C_18_ column (Innoval ODS-2, 5 μM, 19 × 250 mm, Agela Technologies, China). Normal-phase HPLC was performed using a chiralpak IH column (5 μm, 4.6 × 250 mm, Daicel, China). Nuclear magnetic resonance (NMR) spectroscopy was performed using a Bruker AV-600 spectrometer (Billerica, Massachusetts, United States). Optical rotation measurements were obtained using an Autopol IV polarimeter (Rudolph Research Analytical, New Jersey, United States). ECD spectra were performed on a CD-MDS 450 spectrophotometer (JASCO Corporation, Tokyo, Japan).

### 2.2 Plant material

The pseudobulbs of *O. patens* were obtained from Kunming Zhifen Co., Ltd., China, in 2021. Plant identification was verified by Prof. Xiaobo Wang at Dalian Medical University. A voucher specimen (No. 202100401) has been deposited at the College of Integrative Medicine, Dalian Medical University.

### 2.3 Extraction and isolation

The pseudobulbs of *O. patens* (500 g) were extracted with 95% aqueous ethanol (3 × 2 L). The extract (9.8 g) was suspended in 1 L of H_2_O and extracted three times with an equal volume of EtOAc. The EtOAc extract (2.3 g) was subjected to silica gel chromatography using petroleum ether-acetone (1:0–1:1 *v*/*v*) to obtain three fractions, Fr. 1–3. Fr. 1 (25.4 mg) was subjected to preparative HPLC (CH_3_OH-H_2_O, 65:35) using a C_18_ column (Innoval ODS-2, 5 μm, 19 × 250 mm, Agela Technologies, China) and displayed at 210 nm. to give **5** (3.2 mg, *t*_R_ = 15.8 min) and **6** (2.1 mg, *t*_R_ = 25.6 min). Fr. 2 (27.9 mg) was separated by preparative HPLC (CH_3_CN-H_2_O, 60:40) to give **1** (11.5 mg, *t*_R_ = 22.4 min) and **4** (3.3 mg, *t*_R_ = 35.6 min). Fraction 3 (40.9 mg) was prepared by HPLC (CH_3_CN-H_2_O, 65:35) to give **2** (2.3 mg, *t*_R_ = 22.5 min) and **3** (9.0 mg, *t*_R_ = 39.8 min). The isolation of atropisomers **1a**/**1b** (5.0 mg) was performed on HPLC equipped with a chiralpak IH column using *n*-hexane/ethanol (*v*/*v*, 2:1) as mobile phase, resulting in **1a** (1.8 mg, *t*_R_ = 10.2 min) and **1b** (2.1 mg, *t*_R_ = 29.8 min). Atropisomers **2a**/**2b** (2.0 mg), **3a**/**3b** (3.0 mg), and **4a**/**4b** (2.0 mg) were also isolated by HPLC using a chiralpak IH column in *n*-hexane/ethanol (*v*/*v*, 2:1) mobile phase, resulting in **2a** (0.9 mg, *t*_R_ = 19.8 min) and **2b** (0.8 mg, *t*_R_ = 25.6 min), **3b** (1.3 mg, *t*_R_ = 26.9 min) and **3a** (1.2 mg, *t*_R_ = 36.9 min), **4a** (1.0 mg, *t*_R_ = 16.8 min) and **4b** (0.9 mg, *t*_R_ = 28.2 min).

*(*a*S)-2,7,2′-Trihydroxy-4,4′,7′-trimethoxy-1,1′-biphenanthrene (**1a**)* Yellow oil. ${\left[\alpha \right]}_{D}^{20}$ −52 (*c* 0.1, CH_3_OH); ECD (CH_3_OH, Δ*ε*) *λ*_max_ 262 (+9.4), 372 (+3.1) nm.

*(*a*R)-2,7,2′-Trihydroxy-4,4′,7′-trimethoxy-1,1′-biphenanthrene (**1b**)* Yellow oil. ${\left[\alpha \right]}_{D}^{20}$ +55 (*c* 0.1, CH_3_OH); ECD (CH_3_OH, Δ*ε*) *λ*_max_ 266 (−11.8), 317 (+1.2), 371 (−6.0) nm.

*(*a*S)-Phochinenin B (**2a**)* Brown amorphous powder. ${\left[\alpha \right]}_{D}^{20}$ −38 (*c* 0.1, CH_3_OH); ECD (CH_3_OH, Δ*ε*) *λ*_max_ 267 (+1.2), 292 (−0.2), 364 (+0.2) nm.

*(*a*R)-Phochinenin B (**2b**)* Brown amorphous powder. ${\left[\alpha \right]}_{D}^{20}$ +40 (*c* 0.1, CH_3_OH); ECD (CH_3_OH, Δ*ε*) *λ*_max_ 266 (−0.9), 296 (+1.7), 385 (−0.4) nm.

*(*a*S)-4,4′,7,7′-Tetrahydroxy-2,2′-dimethoxy-1,1′-biphenanthrene (**3a**)* Yellow oil. ${\left[\alpha \right]}_{D}^{20}$ −26 (*c* 0.1, CH_3_OH); ECD (CH_3_OH, Δ*ε*) *λ*_max_ 255 (+11.0), 318 (−1.3) nm.

*(*a*R)-4,4′,7,7′-Tetrahydroxy-2,2′-dimethoxy-1,1′-biphenanthrene (**3b**)* Yellow oil. ${\left[\alpha \right]}_{D}^{20}$ +23 (*c* 0.1, CH_3_OH); ECD (CH_3_OH, Δ*ε*) *λ*_max_ 260 (−10.6), 291 (+1.7) nm.

*(*a*S)-Blestriarene C (**4a**)* Yellow oil. ${\left[\alpha \right]}_{D}^{20}$ −46 (*c* 0.1, CH_3_OH); ECD (CH_3_OH, Δ*ε*) *λ*_max_ 260 (+4.7), 319 (−2.6) nm.

*(*a*R)-Blestriarene C (**4b**)* Yellow oil. ${\left[\alpha \right]}_{D}^{20}$ +44 (*c* 0.1, CH_3_OH); ECD (CH_3_OH, Δ*ε*) *λ*_max_ 259 (−10.4), 319 (+3.3) nm.

### 2.4 ECD calculations

Conformational searches were performed using the Conformer Rotamer Ensemble Sampling Tool (18). Geometry optimizations were performed with Gaussian 16 at the B3LYP-D3(BJ)/6-31G(d) level. ECD spectra were then calculated using the TDDFT method at the CAM-B3LYP/def-tzvp level.

### 2.5 Cell culture and cell viability assay

The MH-S cell line was purchased from ATCC (Cat. No. CRL-2019). MH-S macrophages were cultured in RPMI 1640 and incubated at 37 °C with 5% CO_2_. Cytotoxicity was assessed using the CCK-8 assay following 48 h of incubation with the test compounds (19).

### 2.6 Cellular thermal shift assay

MH-S cell lysis was incubated with DMSO or 20 μM compound **3** for 1 h at room temperature, and heated over a gradient of 42–67 °C for 3 min. The cell lysis was clarified by centrifugation at 12,000 rpm (TRUKING, China) at 4 °C for 15 min. The supernatant was analyzed *via* immunoblotting (8).

### 2.7 Drug affinity responsive target stability

Whole cell proteins were isolated by ice-cold buffer containing 1% protease inhibitors (ThermoFisher Scientific, United States). TNC buffer was used to dilute protein solutions. Then, protein solutions were treated with compound **3** (0, 1, 5, 10, and 20 μM) for 1 h at room temperature. Subsequently, pronase was used to react with protein solutions for another 20 min at room temperature. Protein loading buffer was added to stop the reactions, and western blotting was performed (20).

### 2.8 Molecular docking

Discovery Studio software (version 2019; Accelrys, CA, United States) was used for molecular docking. The structure of AMPK was obtained from PDB (ID: 8BIK),^1^ the protein structure was prepared by adding hydrogen atoms and removing water molecules, and then assigned CHARMm force field. A maximum of 20 conformations was generated. The 3D structures of compound **3** and lusianthridin were subjected to energy minimization with the CHARMm force field using the “Prepare Ligand” procedure. The interactions of small molecules within the protein-ligand complex were analyzed using the CDOCKER (21). The maximum number of poses per ligand was set to 30 with a minimum RMSD between final poses of 0.50 Å. The kinetic optimization temperature is 1,000 degrees Celsius, and the optimization steps are 10,000 steps. The non-bond interaction was calculated based on the lattice technique, and the other parameters were the default values of the system.

### 2.9 Molecular dynamics

Molecular dynamics simulations were performed with GROMACS 2021 using the AMBER99SB force field according to previous literature (22, 23). The ligand’s restrained electrostatic potential charges were calculated using Gaussian 16 at the B3LYP/6-31G* level. The initial ligand topology was then generated with Antechamber and ACPYPE. Ligand-protein complexes from docking were centered in a 1.2 nm rectangular box and solvated with a TIP3P water cap. Na^+^ ions were added as counterions to neutralize the system. The complexes underwent steepest descent energy minimization to relax the structure, followed by NVT and NPT equilibration at 300 K and 1 bar, respectively. In the final step, a 100 ns molecular dynamics simulation was conducted at 300 K and 1 bar to analyze interactions between the enzyme and ligand. The trajectory file was processed using GROMACS analysis tools to calculate root mean square deviation (RMSD), root mean square fluctuations (RMSF), and other metrics. Protein-ligand interactions were examined with Discovery Studio, while graphs were generated using GraphPad Prism.

### 2.10 Western blotting

The Western blotting protocol was based on that used in a previous study (24). Briefly, cell and tissue samples were washed three times in chilled phosphate-buffered saline (PBS) before lysis in a specialized buffer. Samples containing 30–40 μg of protein were analyzed using sodium dodecyl sulfate–polyacrylamide gel electrophoresis (SDS–PAGE; 10% gel).

### 2.11 Immunofluorescence

Confocal images were acquired using an Olympus FV3000. The immunofluorescence detection methods for cell and tissue sections were based on previously reported methods (25).

### 2.12 Statistical analysis

For comparisons among multiple groups, the one-way analysis of variance (ANOVA) was performed, followed by Tukey’s *post-hoc* test for multiple comparison adjustments. Statistical significance was defined as *p* < 0.05. All statistical analyses and graph generation were performed using GraphPad Prism 10.0 (GraphPad Software, San Diego, CA, United States).

## 3 Results and discussion

### 3.1 Structure elucidation of isolated compounds

**Figure 2:**
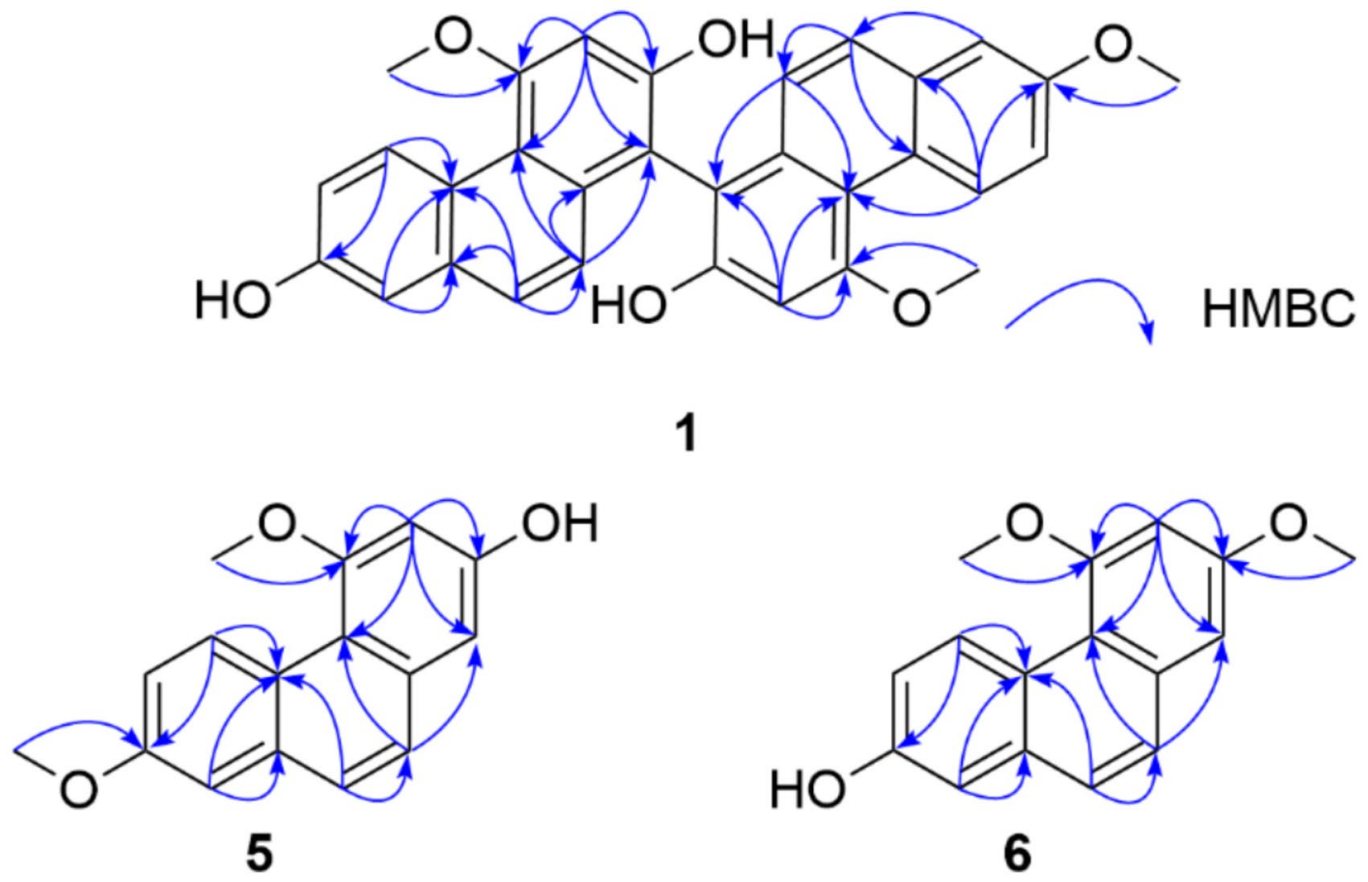
Key HMBC correlations of compounds **1**, **5,** and **6**.

The ^1^H NMR spectrum of compound **1** displayed two sets of phenanthrene signals (Table 1), and the ^13^C NMR spectrum showed a total of 31 carbons, suggesting that compound **1** is a dimeric phenylethylene derivative. The planar structure of compound **1** was established as 2,7,2′-trihydroxy-4,4′,7′-trimethoxy-1,1′-biphenanthrene based on heteronuclear multiple-bond correlations (HMBC) (Figure 2) and comparison with previously reported data (26). In addition, compound **2** was identified as a dimeric phenylethylene derivative based on its ^1^H and ^13^C NMR findings. Its structure was confirmed to be identical to that of phochinenin B, as compared with previously published data (27). In the analysis of compound **3**, the ^1^H and ^13^C NMR spectra revealed only a single set of phenanthrene signals (Table 2), while high-resolution electrospray ionization mass spectrometry (HRESIMS) suggested that compound **3** was a dimer composed of two identical blocks. The structure of compound **3** was subsequently confirmed to be 4,4′,7,7′-tetrahydroxy-2,2′-dimethoxy-1,1′-biphenanthrene (28). Compound **4** possessed the same molecular formula as compound **3**, as supported by its one-dimensional (1D) NMR data (Table 2). Therefore, compound **4** was presumed to be an isomer of compound **3**. When referring to the literature, compound **4** was identified as blestriarene C (29). Compounds **5** and **6** were separated simultaneously by HPLC, with different retention times. Furthermore, they had identical HRESIMS peaks, suggesting that compounds **5** and **6** are also isomers. Given their similar 1D NMR spectra (Table 3), two-dimensional (2D) NMR was performed (Figure 2). When combined with previous literature, compounds **5** and **6** were identified as 2-hydroxy-4,7-dimethoxyphenanthrene (26) and 2,3-dimethoxy-7-hydroxyphenanthrene (30), respectively.

**Table 1:** ^1^H (600 MHz) and ^13^C (150 MHz) of **1** and **2** in DMSO-*d*_6_ (*δ* in ppm, *J* in Hz).

Position	**1**		**2**	
	*δ*_H_ (*J* in Hz)	*δ*_C_	*δ*_H_ (*J* in Hz)	*δ*_C_
1		111.0		115.0
2		153.7	6.66, s	99.3
3	7.01, s	99.7		158.8
4		157.9		156.1
4a		114.3		142.3
4b		123.6		140.8
5	9.39, d (9.3)	128.8	8.10, d (8.7)	130.5
6	7.12, dd (9.3, 2.7)	116.7	6.65, dd (8.7, 2.7)	113.5
7		153.4		156.0
8	7.07, d (2.7)	111.1	6.58, d (2.7)	114.7
8a		132.5		126.5
9	7.32, d (9.2)	126.8	2.30, m; 2.16, m	31.0
10	6.92, d (9.2)	124.8	2.51, m; 2.45, m	28.3
10a		133.4		117.7
1′		111.0		112.3
2′		154.2	6.93, s	100.2
3′	7.03, s	99.8		160.2
4′		157.8		154.0
4a′		114.1		134.9
4b′		124.6		134.5
5′	9.39, d (9.5)	128.8	9.45, d (9.1)	130.3
6′	7.12, dd (9.3, 2.7)	116.2	7.10, dd (9.1, 2.9)	117.3
7′		156.0		154.0
8′	7.07, d (2.7)	108.4	7.11, d (2.9)	112.0
8a′		132.3		125.8
9′	6.99, d (9.2)	127.1	7.42, d (9.1)	128.4
10′	6.92, d (9.2)	125.1	7.20, d (9.1)	125.6
10a′		133.7		116.8
3-OCH_3_			3.95, s	56.0
4-OCH_3_	4.12, s	55.5		
3′-OCH_3_			4.15, s	56.0
4′-OCH_3_	4.13, s	55.6		
7′-OCH_3_	3.86, s	55.0		

**Table 2:** ^1^H (600 MHz) and ^13^C (150 MHz) of **3** and **4** in DMSO-*d*_6_ (*δ* in ppm, *J* in Hz).

Position	**3**		**4**	
	*δ*_H_ (*J* in Hz)	*δ*_C_	*δ*_H_ (*J* in Hz)	*δ*_C_
1, 1′		111.5		111.1
2, 2′		160.5		153.4
3, 3′	7.01, s	100.3	6.99, s	99.7
4, 4′		155.4		157.8
4a, 4a′		117.0		114.3
4b, 4b′		125.8		123.6
5, 5′	9.49, d (9.4)	130.5	9.37, d (9.4)	128.9
6, 6′	7.12, dd (9.4, 2.8)	112.0	7.10, dd (9.4, 2.7)	116.7
7, 7′		154.6		154.2
8, 8′	7.08, d (2.8)	117.3	7.06, d (2.7)	111.0
8a, 8a′		135.4		132.5
9, 9′	7.31, d (9.2)	128.4	7.30, d (9.2)	126.8
10, 10′	7.01, d (9.2)	125.9	6.91, d (9.2)	124.8
10a, 10a′		134.6		133.4
2, 2′-OCH_3_	4.18, s	56.0		
4, 4′-OCH_3_			4.11, s	55.6

**Table 3:** ^1^H (600 MHz) and ^13^C (150 MHz) of **5** and **6** in DMSO-*d*_6_ (*δ* in ppm, *J* in Hz).

Position	**5**		**6**	
	*δ*_H_ (*J* in Hz)	*δ*_C_	*δ*_H_ (*J* in Hz)	*δ*_C_
1	6.85, br s	104.5	7.03, d (2.4)	101.7
2		155.0		157.1
3	6.77, d (2.3)	99.7	6.83, d (2.4)	99.3
4		158.8		158.6
4a		113.5		115.0
4b		124.3		123.0
5	9.31, d (9.4)	128.5	9.27, d (9.2)	128.7
6	7.19, dd (9.4, 2.5)	116.2	7.10, dd (9.2, 2.6)	116.8
7		156.0		154.5
8	7.37, d (2.5)	108.8	7.19, d (2.6)	111.3
8a		132.6		133.1
9	7.67, d (8.8)	127.5	7.64, d (8.8)	127.1
10	7.58, d (8.8)	127.1	7.61, d (8.8)	127.5
10a		134.1		133.5
2-OCH_3_			3.89, s	55.2
4-OCH_3_	4.03, s	55.6	4.05, s	55.8
7-OCH_3_	3.88, s	55.0		

### 3.2 Optical resolution and stereochemical establishment of atropisomers

**Figure 3:**
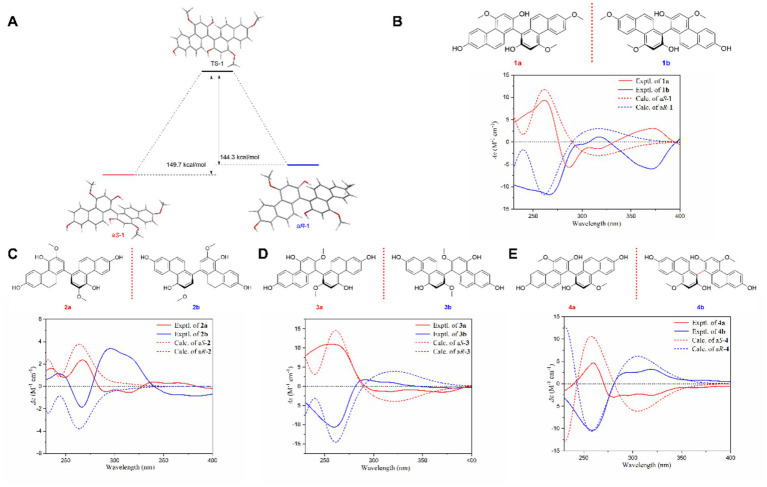
Optical resolution and stereochemical assignment of atropisomeric phenanthrene dimers **1**–**4**. **(A)** Tautomerism analysis of atropisomeric compounds (a*S*)-**1** and (a*R*)-**1**. **(B)** Optical resolution and stereochemical assignment of **1a** and **1b**. **(C)** Optical resolution and stereochemical assignment of **2a** and **2b**. **(D)** Optical resolution and stereochemical assignment of **3a** and **3b**. **(E)** Optical resolution and stereochemical assignment of **4a** and **4b**.

Considering that the two units of the dimeric compounds **1**–**4** are linked *via* carbon–carbon bonds at C-1 and C-1′, it is reasonable to speculate that these compounds may exist as tautomers due to steric hindrance. Indeed, some dimeric phenanthrene analogues exhibit atropisomerism, which can be stable under natural conditions and separated by chiral chromatography. Examples include bletistriatins A and B from *Bletilla striata* (Thunb. ex A. Murray) Reichenbach f. (31). We initially employed computational chemistry using density functional theory (DFT) to calculate the potential interconversion between two hypothetical atropisomers of compound **1**, a*S*-1 and a*R*-1 (Figure 3A). The results showed that a*S*-1 and a*R*-1 possess similar molecular energies, and that the transition state of the interconversion reaction, TS-1, in which the two monomeric phenanthrene units are coplanar, presents barriers exceeding 100 kcal/mol for both isomers. These results suggest that the two atropisomers of compound **1** are stable at room temperature (32). Accordingly, normal-phase chiral chromatography was performed on dimers **1**–**4**, which resulted in four pairs of enantiomers, **1a**/**1b**–**4a**/**4b**. The enantiomeric nature of these compounds was confirmed by mirror-image ECD curves (Figures 3B–E). Furthermore, their absolute configurations were established by comparing experimental and calculated ECD spectra. A total of 10 phenanthrene derivatives, including four pairs of dimeric atropisomers and one pair of monomeric isomers, were obtained from *O. patens* for the first time. Three pairs of them, compounds **1a/1b**, **2a/2b**, and **4a/4b**, possess unreported absolute configurations, suggest that the biosynthesis of phenanthrene dimers in *O. patens* lacks stereoselectivity.

### 3.3 Anti-inflammatory effects of compounds **1–6**

**Figure 4:**
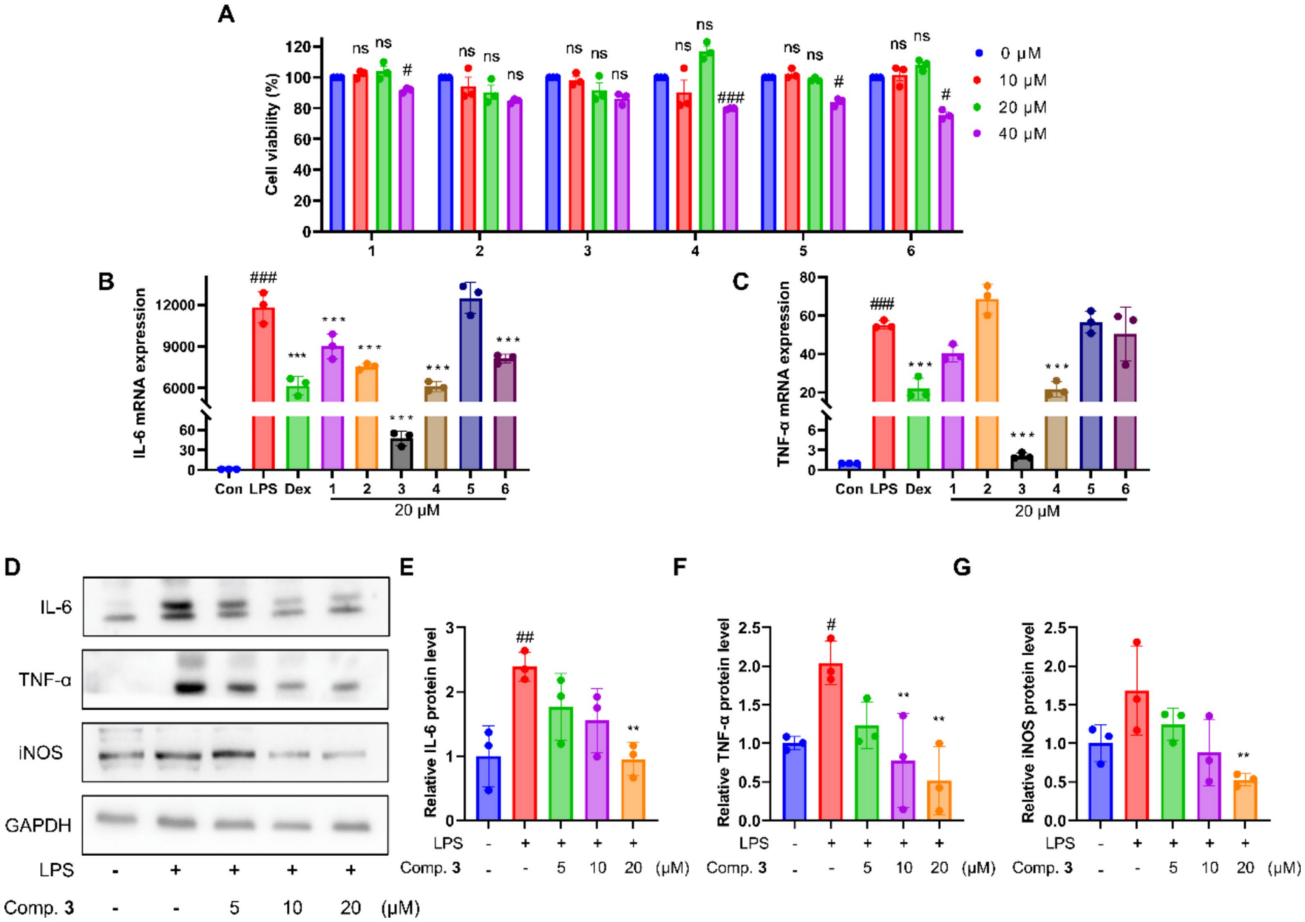
Phenanthrene dimer **3** exhibits anti-inflammatory effects on LPS-stimulated macrophage MH-S cells. **(A)** Cytotoxicity of compounds **1**–**6** against MH-S cells. **(B)** Inhibitory effects of compounds **1**–**6** on LPS-induced IL-6 transcription production in MH-S cells. **(C)** Inhibitory effects of compounds **1**–**6** on LPS-induced TNF-*α* transcription production in MH-S cells. **(D–G)** The effects of compound **3** treatment on inflammatory cytokine TNF-α, IL-6, and iNOS protein expressions of LPS-induced MH-S cells. Data are presented as mean ± SD (*n* = 3, biological replicates); *p < 0.05, **p < 0.01, and ***p < 0.001 vs. LPS; ^#^*p* < 0.05, ^##^*p* < 0.01, and ^###^*p* < 0.001 vs. Con.

As a functional food, the pseudobulbs of *O. patens* are used treat a variety of inflammatory diseases. Inspired by this, a cell model of inflammation was established using LPS-induced MH-S macrophages and used to evaluate the anti-inflammatory potential of compounds **1**–**6**. First, their cytotoxicity against MH-S cells was evaluated. As shown in Figure 4A, compounds **1**–**6** did not exhibit effects on cell viability at concentrations lower than 20 μM. Compounds **3** and **4** significantly inhibited the transcriptional level of typical inflammatory factors, IL-6 and TNF-*α*, without affecting cell viability (Figures 4B,C). Among them, compound **3** possessed the most outstanding activity comparable to the positive control, dexamethasone (Dex). Moreover, compound **3** inhibited inflammatory factor protein secretion, including IL-6, TNF-α, and iNOS at 20 μM (Figures 4D–G). In addition, compounds **1–6** were screened for their anti-inflammatory activity using NO production as an indicator in LPS-stimulated RAW264.7 macrophage (Supplementary Figure S2). The results showed that compounds **1** and **3** significantly inhibited LPS-induced NO production at 20 μM.

### 3.4 Compound **3** exerted anti-inflammatory effects by directly activating AMPK

**Figure 5:**
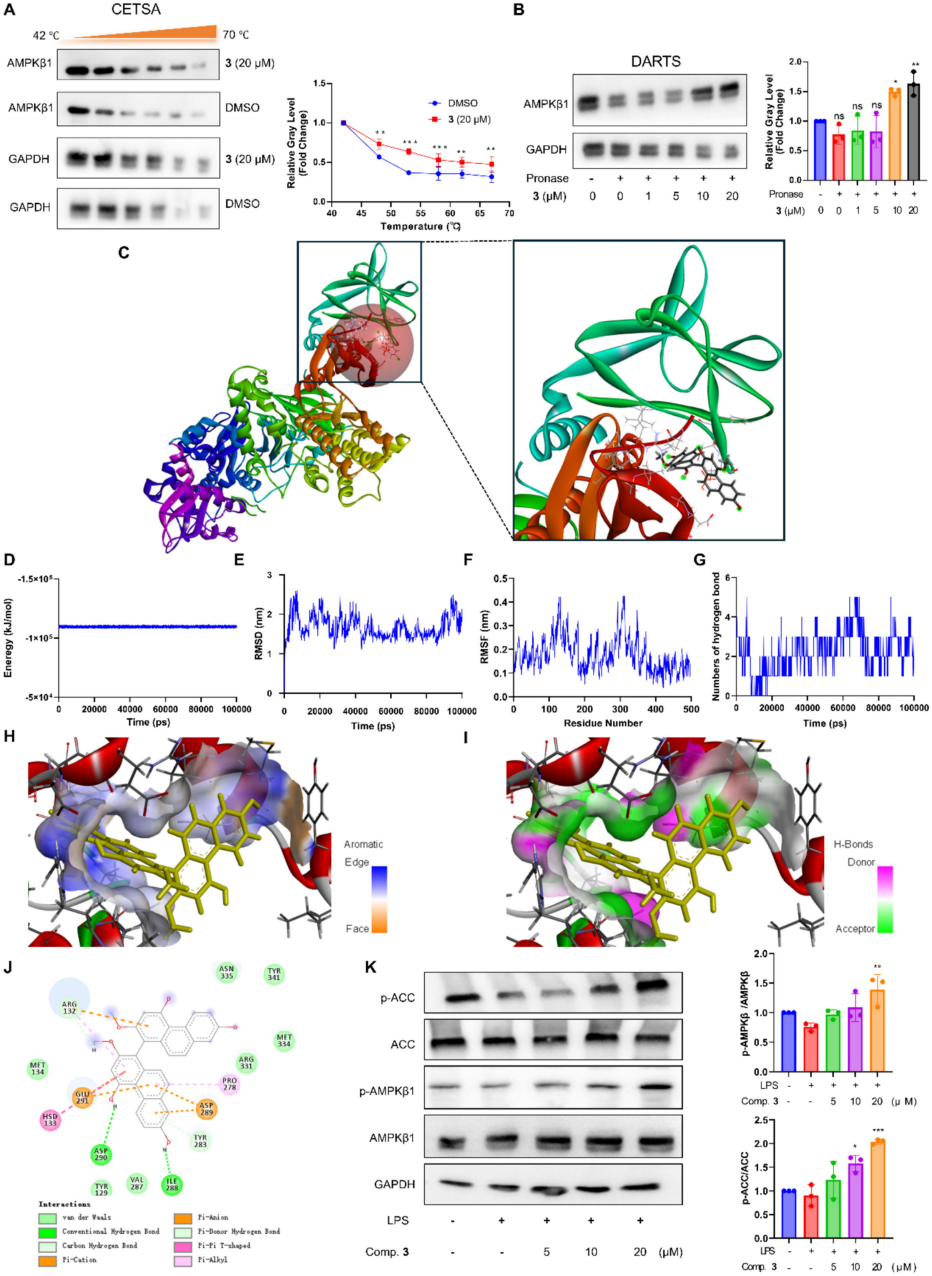
Compound **3** activated AMPK in MH-S cells. **(A)** Detection of the interaction between compound **3** and AMPK by CETSA. **(B)** Detection of the interaction between compound **3** and AMPK by DARTS; data are presented as mean ± SD (*n* = 3); ^*^p < 0.05, **p < 0.01, and ***p < 0.001 vs. DMSO. **(C)** Compound **3** could enter into the ADaM binding site on AMPK. **(D)** The potential energy of compound **3** with AMPK in 100 ns of molecular dynamics simulation. **(E)** RMSD of compound **3** with AMPK in 100 ns of molecular dynamics simulation. **(F)** RMSF of compound **3** with AMPK in 100 ns of molecular dynamics simulation. **(G)** The number of hydrogen bonds in 100 ns of molecular dynamics simulation. **(H)** Aromatic binding interface between compound **3** and AMPK at 100th ns of molecular dynamics simulation. **(I)** Hydrogen bonding interface between compound **3** and AMPK at 100th ns of molecular dynamics simulation. **(J)** The interactions of compound **3** with AMPK at 100th ns of molecular dynamics simulation; **(K)** The effects of compound **3** treatment on AMPK and ACC phosphorylation levels in MH-S cells; *p < 0.05, ^**^p < 0.01, and ***p < 0.001 vs. LPS; ^#^*p* < 0.05, ^##^*p* < 0.01, and ^###^*p* < 0.001 vs. Con.

Given that compound **3** exhibited significant anti-inflammatory effects, we further investigated the potential mechanism. Lusianthridin, along with other structurally related phenanthrene analogues, has been demonstrated to directly bind to AMPK, thereby activating downstream pathways (33). Taking into account the structural similarity between compound **3** and lusianthridin, we investigated whether compound **3** could exert its effects through a similar mechanism. We employed both cellular thermal shift assay (CETSA) and drug affinity responsive target stability (DARTS) to determine whether compound **3** could stably bind to AMPK in MH-S cells (8, 20). As shown in Figures 5A,B, in the presence of compound **3**, the AMPK protein exhibited significant resistance to destabilization induced by temperature and protease, suggesting that compound **3** can stably bind to AMPK within MH-S cells. It was reported that the activators of AMPK from natural plant compounds bind to the allosteric drug and metabolite (ADaM)–binding site of AMPK (33, 34). Therefore, molecular docking based on computational biology was employed to investigate the interaction between compound **3** and the AMPK protein complex, using known AMPK activator lusianthridin as a control (Supplementary Figure S1). As shown in Figure 5C, similar to lusianthridin, compound **3** can readily enter the ADaM site of the AMPK complex. To further validate the dynamic stability and binding mode of compound **3** with AMPK, we performed 100 ns molecular dynamics (MD) simulations. During 100 ns of MD simulation, the energy of the complex of compound **3** with AMPK was about −1 × 10^5^ kJ/mol (Figure 5D), although higher than the reference complex (−1 × 10^6^ kJ/mol) (Supplementary Figure S1B), it also demonstrates that compound **3** can stably bind to AMPK. As shown in Figure 5E, the Root Mean Square Deviation (RMSD) of the compound **3**-AMPK complex reached a stable plateau after approximately 20 ns, with an average deviation of 1.5 nm. Compared to the lusianthridin-AMPK complex (Supplementary Figure S1C), the convergence of the RMSD trajectory confirms that Compound **3** maintains a highly stable and equilibrated conformation throughout the simulation. The Root Mean Square Fluctuation (RMSF) analysis (Figure 5F) revealed that the residues comprising the ADaM binding pocket exhibited significantly low flexibility. Notably, in the region of residues 100–150, Compound **3** effectively “locked” the protein architecture, resulting in RMSF values lower than 0.2 nm, which is comparable to, or even more rigid than, those of the positive control lusianthridin (Supplementary Figure S1D). Furthermore, we monitored the intermolecular hydrogen bonds during the 100 ns trajectory. Compound **3** exhibited a superior hydrogen-bonding network, maintaining 2–5 consistent hydrogen bonds in the latter 40 ns of the simulation (Figure 5G), whereas lusianthridin fluctuated between 1 and 3 bonds (Supplementary Figure S1E). From a chemical perspective, compound **3** shares some of the same structural features as lusianthridin, including a highly aromatic skeleton and phenolic hydroxyls ready to interact with AMPK. As expected, the interface between compound **3** and AMPK exhibited aromatic and hydrogen bonding interactions at 100th ns of MD simulation (Figures 5H–I). Detailed 2D interaction analysis at 100th ns of MD simulation (Figure 5J) indicated that Compound **3** forms a complex interaction network involving hydrogen bonding with Asp290 and Ile288, and extensive hydrophobic stacking with the aromatic residues in the ADaM site. These findings indicate that compound **3** can form direct and stable interactions with AMPK. We then investigated whether compound **3** could activate AMPK and its downstream signaling pathway. What we found is that compound **3** increased the phosphorylation of AMPKβ1 (p-AMPKβ1), and the substrate (Figure 5K). Collectively, these reuslts confirming that Compound **3** is a potent and stable direct activator of the AMPK.

Based on the combined experimental results, a clear structure–activity relationship (SAR) was established. Firstly, the dimeric phenanthrenes (**1–4**) generally exhibited superior potential compared to their monomeric counterparts (**5** and **6**), suggesting that the increased molecular surface area and the specific spatial orientation provided by the dimer skeleton are essential for effective binding within the large interfacial ADaM site of the AMPK. Among the dimers, compounds **3** and **4** showed significant activity, whereas **1** and **2** were largely inactive. The lack of potency in compound **2** suggests that the saturation of the C_9_–C_10_ bond (dihydrophenanthrene moiety) disrupts the planar aromaticity required for stable ***π*-π** stacking interactions within the binding pocket. Most notably, compound **3** demonstrated the highest potency, outperforming its structural analogs, compounds **1** and **4**. The primary structural difference lies in the substitution at the C_4_ position; compound **3** possesses a free hydroxyl group (-OH), while compounds **1** and **4** are methylated at this position (-OCH₃). As shown in Figure 5J, this specific hydroxyl group in compound **3** is a critical pharmacophore, acting as a hydrogen bond donor to interact with key residues such as Asp290. The methylation in compounds **1** and **4** likely introduces steric hindrance or eliminates this essential polar interaction, leading to reduced affinity.

### 3.5 Compound **3** inhibited LPS-induced NF-κB signaling activation

**Figure 6:**
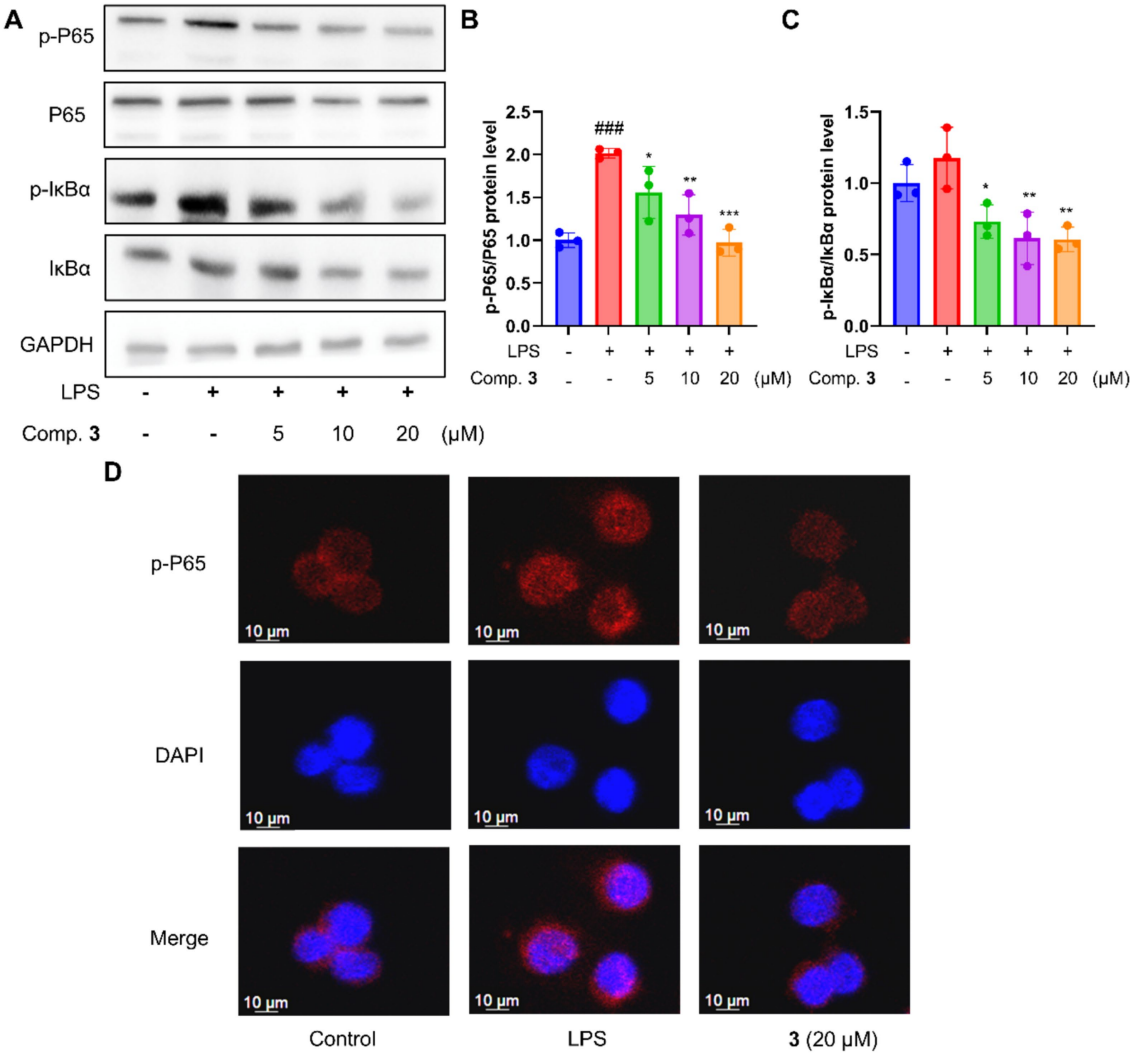
Phenanthrene dimer **3** inhibited LPS-induced NF-κB pathway activation in LPS-stimulated macrophage MH-S cells. **(A)** Effects of compound **3** toward the activation of NF-κB p65; quantitative data of p-P65/P65 **(B)** and p-IκBα/IκBα **(C,D)** Compound **3** prevented LPS-induced nuclear translocation of p65 in MH-S cells. Data are presented as mean ± SD (*n* = 3); *p < 0.05, **p < 0.01, and ***p < 0.001 vs. LPS; ^#^*p* < 0.05, ^##^*p* < 0.01, and ^###^*p* < 0.001 vs. Con.

There is evidence that AMPK activation inhibits the inflammatory response by modulating the NF-κB pathway (12, 35). Therefore, the effects of compound **3** on the NF-κB pathway were explored. As key nodes in NF-κB signaling, the phosphorylation of IκBα and P65 is a marker of NF-κB signaling activation. As shown in Figures 6A–C, compound **3** dose-dependently reduced LPS-induced phosphorylation of IκBα and P65, suggesting that compound **3** can inhibit the activation of NF-κB signaling. The conclusion was further confirmed by immunofluorescence staining (Figure 6D), which showed that compound **3** blocked the phosphorylation of P65 and its nuclear translocation.

## 4 Conclusion

In our metabolomic study of the edible parts of *O. patens*, 10 phenanthrene derivatives, including four pairs of atropisomeric phenanthrene dimers (compounds **1a**/**1b**–**4a**/**4b**) and two monomers (compounds **5** and **6**), were obtained for the first time. More importantly, phenanthrene dimer **3** demonstrated potential anti-inflammatory effects in LPS-stimulated MH-S macrophages. Mechanistic studies indicated that compound **3** can stably bind to the kinase AMPK, thereby activating it, increasing the phosphorylation of downstream ACC, and subsequently regulating the NF-κB signaling to exert anti-inflammatory effects. The report provides a new example of natural plant compounds from dietary sources with anti-inflammatory effects.

## Data Availability

The original contributions presented in the study are included in the article/Supplementary material, further inquiries can be directed to the corresponding authors.

## Footnotes

1
www.rcsb.org
