# Supplementary material for: Discovery of anti-inflammatory agents from Oreorchis patens, a medicinal and edible plant: mechanistic insights and potential therapeutic applications
Source: Front Nutr. 2026 Mar 23;13:1781206. doi: 10.3389/fnut.2026.1781206 (PMC13051381; doi:10.3389/fnut.2026.1781206)
Supplement: Supplementary file 1 [file Supplementary_file_1.docx]

**Supporting information for**

**Discovery of anti-inflammatory agents from *Oreorchis patens*, a medicinal and edible plant: mechanistic insights and potential therapeutic applications**

Fan Wei^1,†^, Ruonan Wang^2,†^, Yiming Liu^3,†^, Hui Ren^3^, Wenwen Yin^3^, Hongyu Sun^3^, Wenyu Zhao^3,^*, Shijun Li^1,^*

^1^ *Department of Clinical Laboratory, The First Affiliated Hospital of Dalian Medical University, Dalian 116011, China*

^2^ *School of Traditional Chinese Materia Medica, Shenyang Pharmaceutical University, Shenyang 110016, China*

^3^ *College (Institute) of Integrative Medicine, Dalian Medical University, Dalian 116044, China*

*Correspondence: Wenyu Zhao, E-mail: [wenyuzhao2019@163.com](mailto:wenyuzhao2019@163.com); Shijun Li, E-mail: lishijun@dmu.edu.cn

^†^ These authors contributed equally to this work.

Table of contents

[**Figure S1.** (A) Lusianthridin in the ADaM binding site of AMPK; (D) The potential energy of lusianthridin with AMPK in 100 ns of molecular dynamics simulation; (**E**) RMSD of lusianthridin with AMPK in 100 ns of molecular dynamics simulation; (**F**) RMSF of lusianthridin with AMPK in 100 ns of molecular dynamics simulation; (**G**) The number of hydrogen bonds in 100 ns of molecular dynamics simulation; (H) Aromatic binding interface between lusianthridin and AMPK at 100th ns of molecular dynamics simulation; (I) Hydrogen bonding interface between lusianthridin and AMPK at 100th ns of molecular dynamics simulation; (J) The interactions of lusianthridin with AMPK at 100th ns of molecular dynamics simulation. 4](#_Toc223599588)

[**Figure S2.** The effects of compounds **1-6** on NO production in LPS-stimulated RAW264.7 cells. **p* < 0.05, ***p* < 0.01, and ****p* < 0.001 *vs* LPS; ^#^*p* < 0.05, ^##^*p* < 0.01, and ^###^*p* < 0.001 *vs* Con. 5](#_Toc223599589)

[**Figure S3.** ^1^H NMR (600 MHz, DMSO-*d*_6_) spectrum of **1**. 6](#_Toc223599590)

[**Figure S4.** ^13^C NMR (150 MHz, DMSO-*d*_6_) spectrum of **1**. 6](#_Toc223599591)

[**Figure S5.** HSQC (600 MHz, DMSO-*d*_6_) spectrum of **1**. 7](#_Toc223599592)

[**Figure S6.** HMBC (600 MHz, DMSO-*d*_6_) spectrum of **1**. 7](#_Toc223599593)

[**Figure S7.** ^1^H NMR (600 MHz, CD_3_OD) spectrum of **2**. 8](#_Toc223599594)

[**Figure S8.** ^13^C NMR (150 MHz, CD_3_OD) spectrum of **2**. 8](#_Toc223599595)

[**Figure S9.** ^1^H NMR (600 MHz, CD_3_OD) spectrum of **3**. 9](#_Toc223599596)

[**Figure S10.** ^13^C NMR (150 MHz, CD_3_OD) spectrum of **3**. 9](#_Toc223599597)

[**Figure S11.** ^1^H NMR (600 MHz, DMSO-*d*_6_) spectrum of **4**. 10](#_Toc223599598)

[**Figure S12.** ^13^C NMR (150 MHz, DMSO-*d*_6_) spectrum of **4**. 10](#_Toc223599599)

[**Figure S13.** ^1^H NMR (600 MHz, DMSO-*d*_6_) spectrum of **5**. 11](#_Toc223599600)

[**Figure S14.** ^13^C NMR (150 MHz, DMSO-*d*_6_) spectrum of **5**. 11](#_Toc223599601)

[**Figure S15.** HSQC (600 MHz, DMSO-*d*_6_) spectrum of **5**. 12](#_Toc223599602)

[**Figure S16.** HMBC (600 MHz, DMSO-*d*_6_) spectrum of **5**. 12](#_Toc223599603)

[**Figure S17.** ^1^H NMR (600 MHz, DMSO-*d*_6_) spectrum of **6**. 13](#_Toc223599604)

[**Figure S18.** ^13^C NMR (150 MHz, DMSO-*d*_6_) spectrum of **6**. 13](#_Toc223599605)

[**Figure S19.** HSQC (600 MHz, DMSO-*d*_6_) spectrum of **6**. 14](#_Toc223599606)

[**Figure S20.** HMBC (600 MHz, DMSO-*d*_6_) spectrum of **6**. 14](#_Toc223599607)

[**Figure S20.** The raw western blot images for Figure 4D. 17](#_Toc223599608)

[**Figure S21.** The raw western blot images for Figure 5G. 20](#_Toc223599609)

[**Figure S22.** The raw western blot images for Figure 6A. 24](#_Toc223599610)


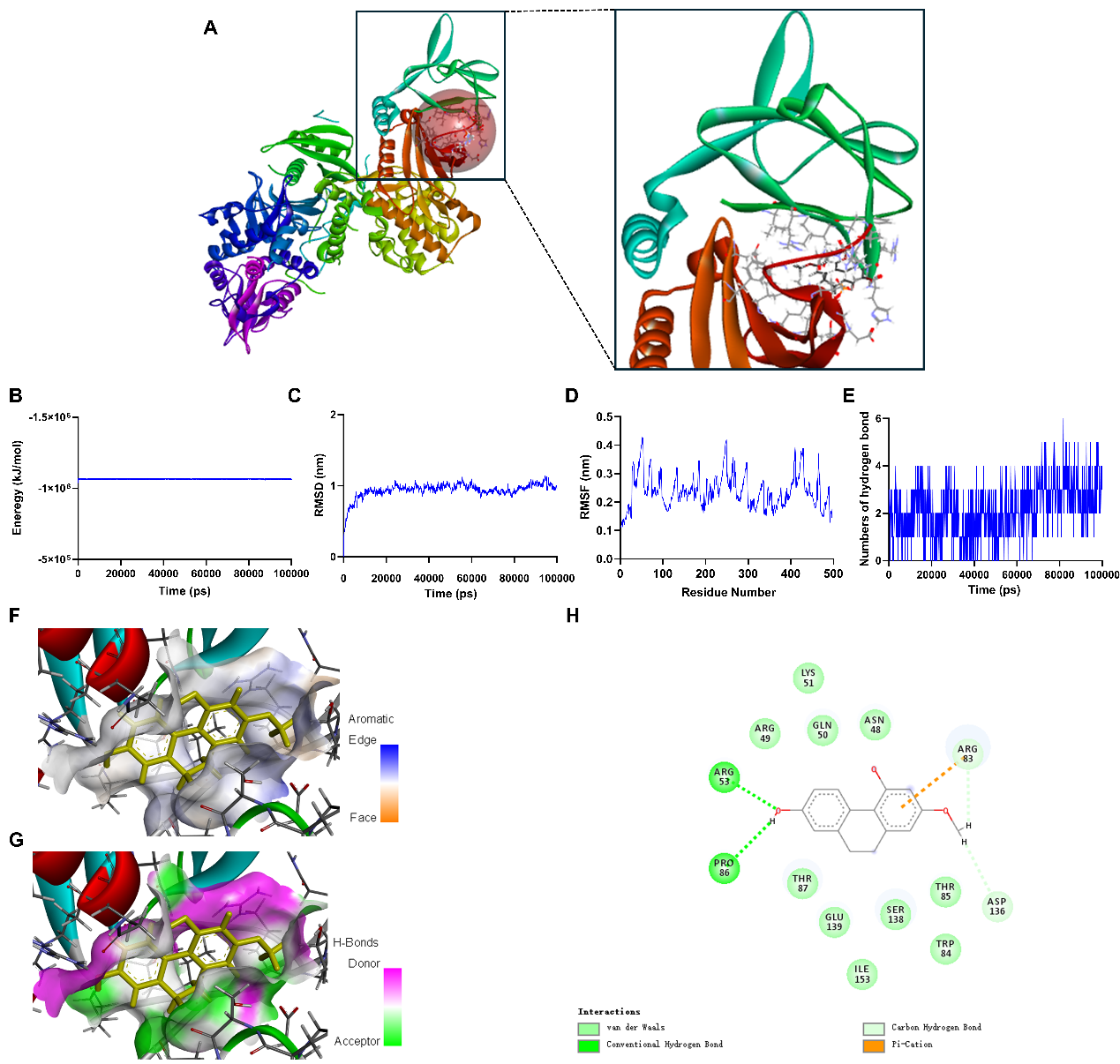


**Figure S1.** (A) Lusianthridin in the ADaM binding site of AMPK; (D) The potential energy of lusianthridin with AMPK in 100 ns of molecular dynamics simulation; (**E**) RMSD of lusianthridin with AMPK in 100 ns of molecular dynamics simulation; (**F**) RMSF of lusianthridin with AMPK in 100 ns of molecular dynamics simulation; (**G**) The number of hydrogen bonds in 100 ns of molecular dynamics simulation; (H) Aromatic binding interface between lusianthridin and AMPK at 100th ns of molecular dynamics simulation; (I) Hydrogen bonding interface between lusianthridin and AMPK at 100th ns of molecular dynamics simulation; (J) The interactions of lusianthridin with AMPK at 100th ns of molecular dynamics simulation.

**Figure S2.** The effects of compounds **1-6** on NO production in LPS-stimulated RAW264.7 cells. **p* < 0.05, ***p* < 0.01, and ****p* < 0.001 *vs* LPS; ^#^*p* < 0.05, ^##^*p* < 0.01, and ^###^*p* < 0.001 *vs* Con.

**Figure S3.** ^1^H NMR (600 MHz, DMSO-*d*_6_) spectrum of **1**.

**Figure S4.** ^13^C NMR (150 MHz, DMSO-*d*_6_) spectrum of **1**.

**Figure S5.** HSQC (600 MHz, DMSO-*d*_6_) spectrum of **1**.

**Figure S6.** HMBC (600 MHz, DMSO-*d*_6_) spectrum of **1**.

**Figure S7.** ^1^H NMR (600 MHz, CD_3_OD) spectrum of **2**.

**Figure S8.** ^13^C NMR (150 MHz, CD_3_OD) spectrum of **2**.

**Figure S9.** ^1^H NMR (600 MHz, CD_3_OD) spectrum of **3**.

**Figure S10.** ^13^C NMR (150 MHz, CD_3_OD) spectrum of **3**.

**Figure S11.** ^1^H NMR (600 MHz, DMSO-*d*_6_) spectrum of **4**.

**Figure S12.** ^13^C NMR (150 MHz, DMSO-*d*_6_) spectrum of **4**.

**Figure S13.** ^1^H NMR (600 MHz, DMSO-*d*_6_) spectrum of **5**.

**Figure S14.** ^13^C NMR (150 MHz, DMSO-*d*_6_) spectrum of **5**.

**Figure S15.** HSQC (600 MHz, DMSO-*d*_6_) spectrum of **5**.

**Figure S16.** HMBC (600 MHz, DMSO-*d*_6_) spectrum of **5**.

**Figure S17.** ^1^H NMR (600 MHz, DMSO-*d*_6_) spectrum of **6**.

**Figure S18.** ^13^C NMR (150 MHz, DMSO-*d*_6_) spectrum of **6**.

**Figure S19.** HSQC (600 MHz, DMSO-*d*_6_) spectrum of **6**.

**Figure S20.** HMBC (600 MHz, DMSO-*d*_6_) spectrum of **6**.


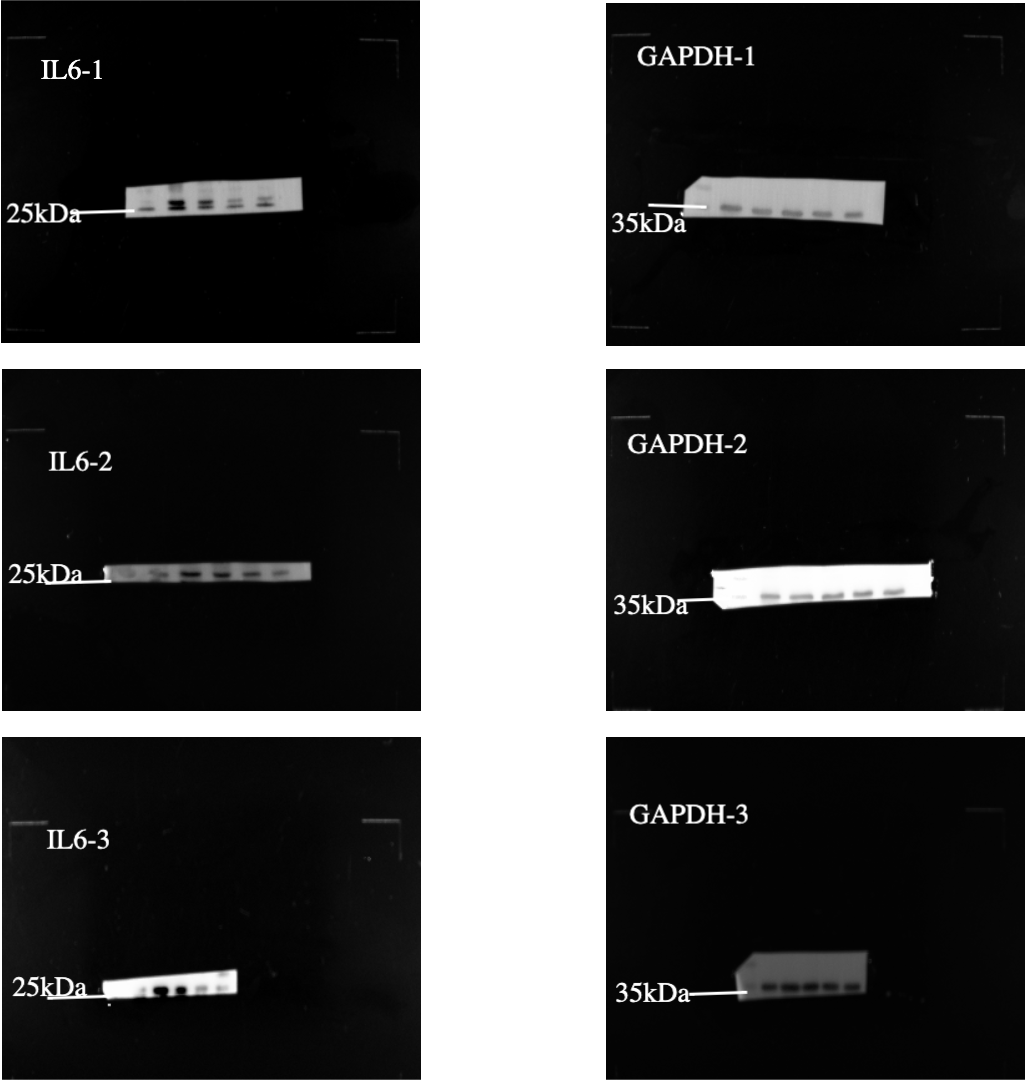


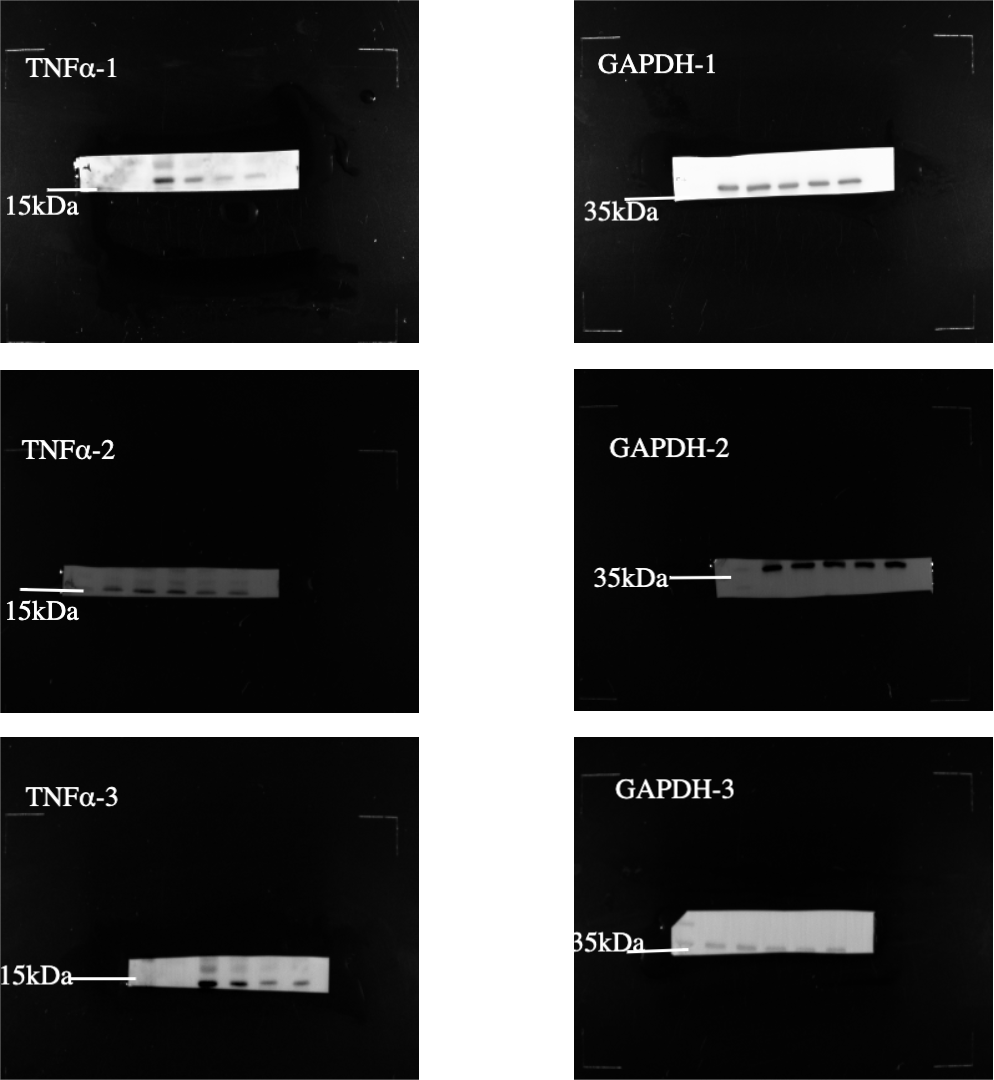


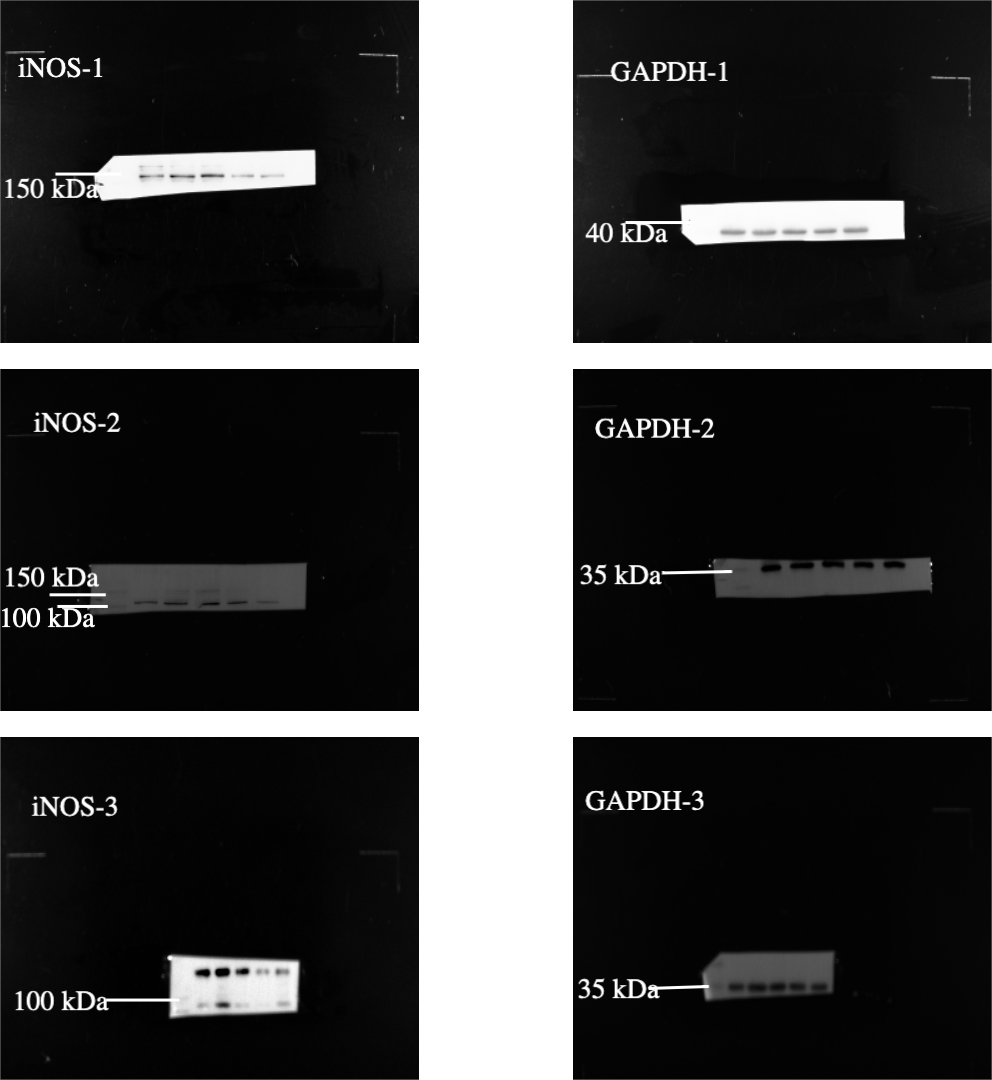


**Figure S20.** The raw western blot images for Figure 4D.


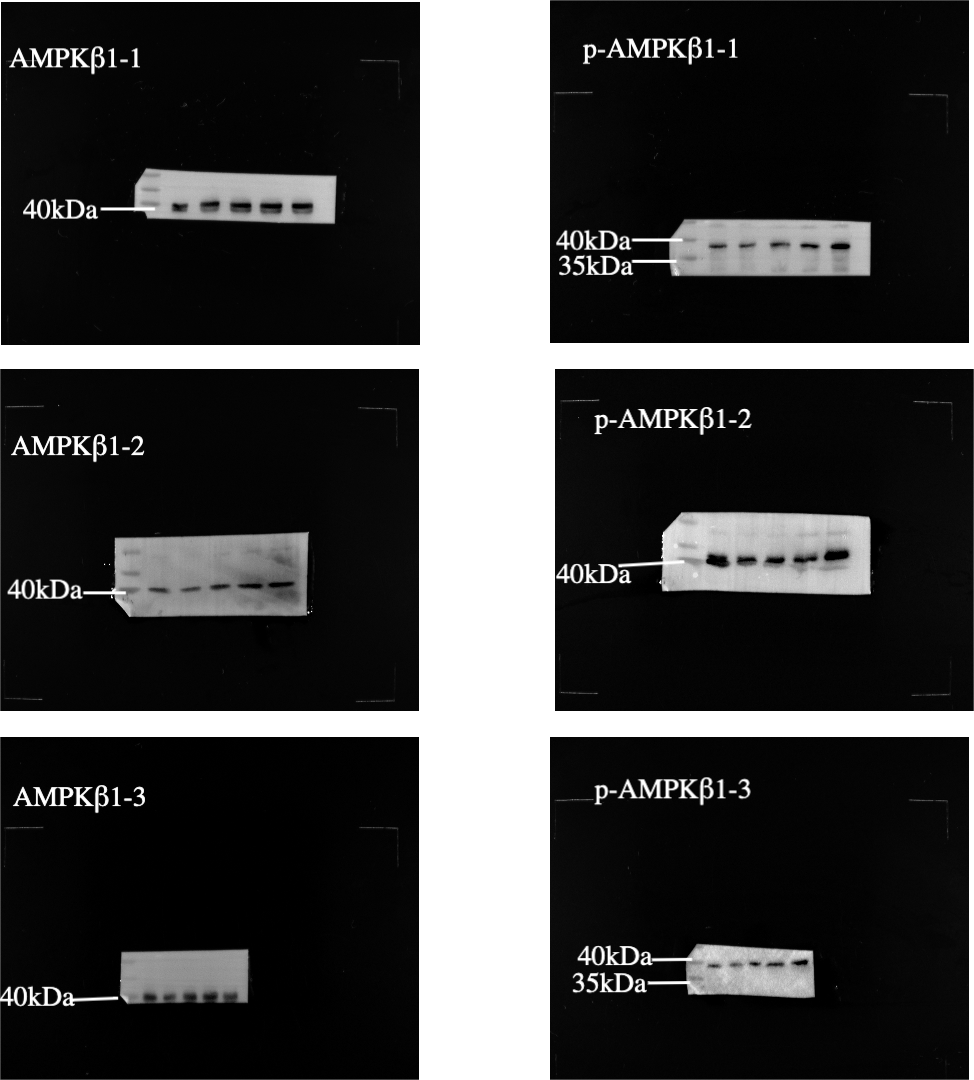


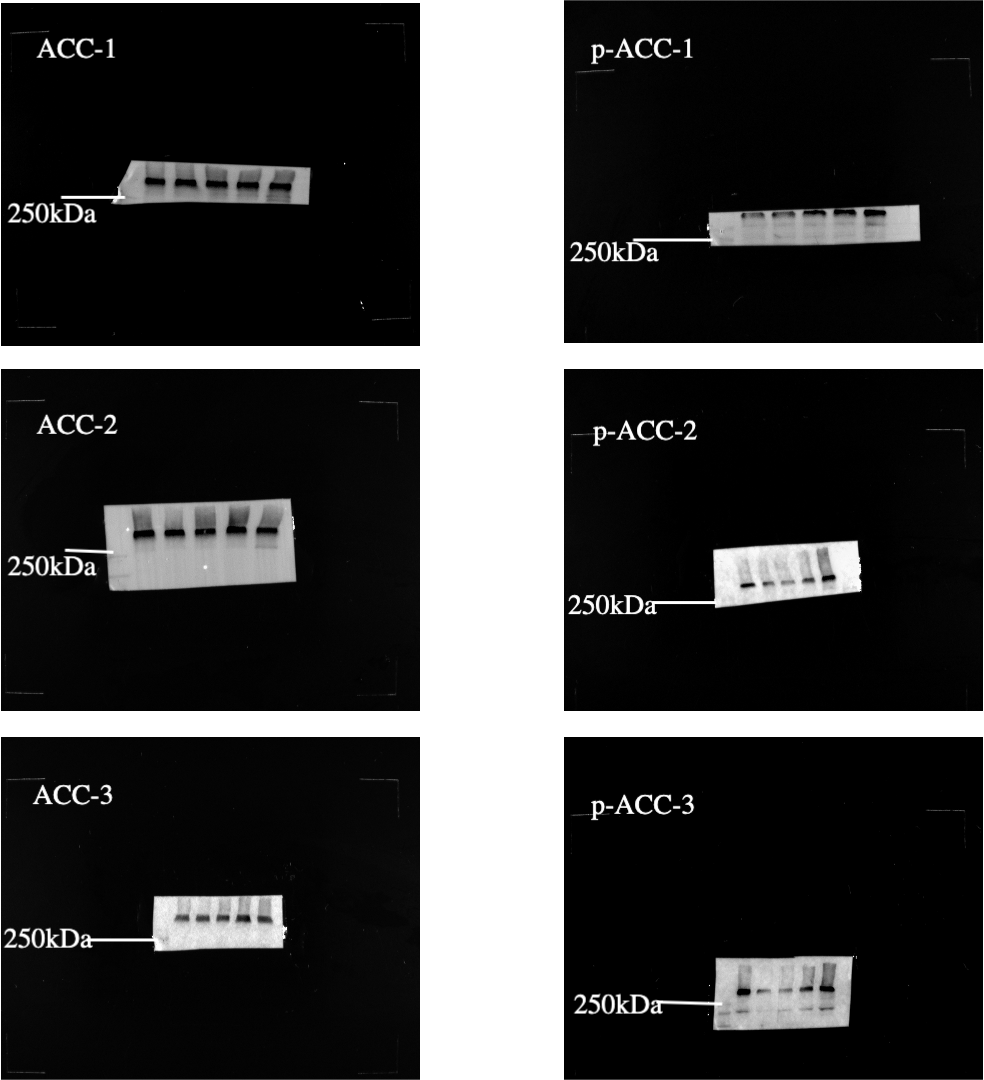


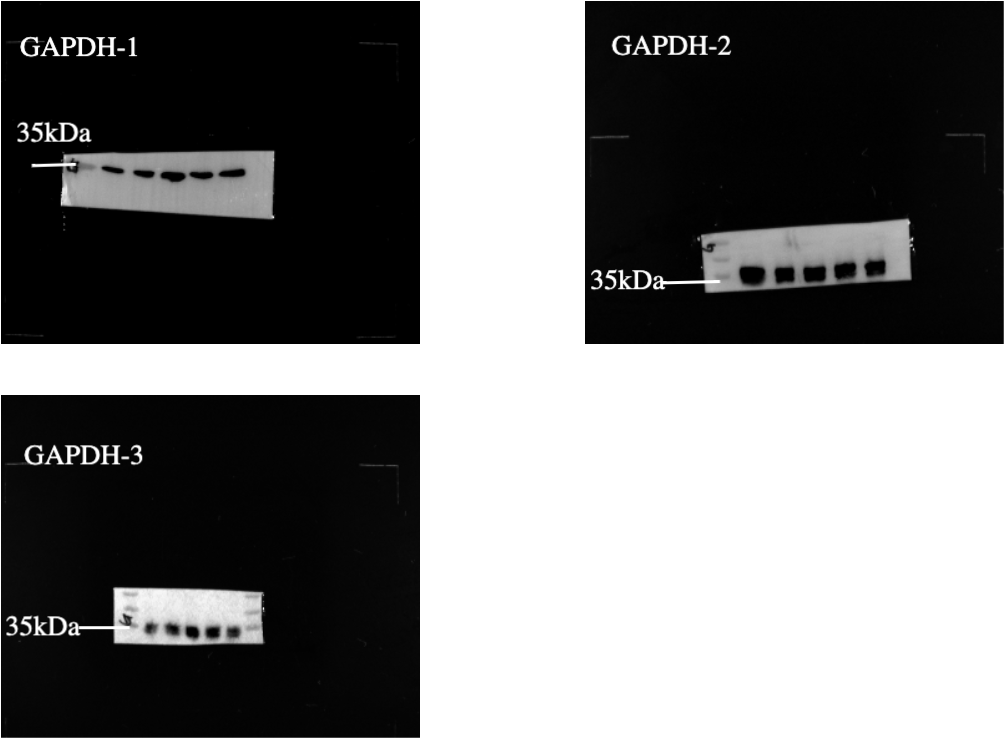


**Figure S21.** The raw western blot images for Figure 5G.


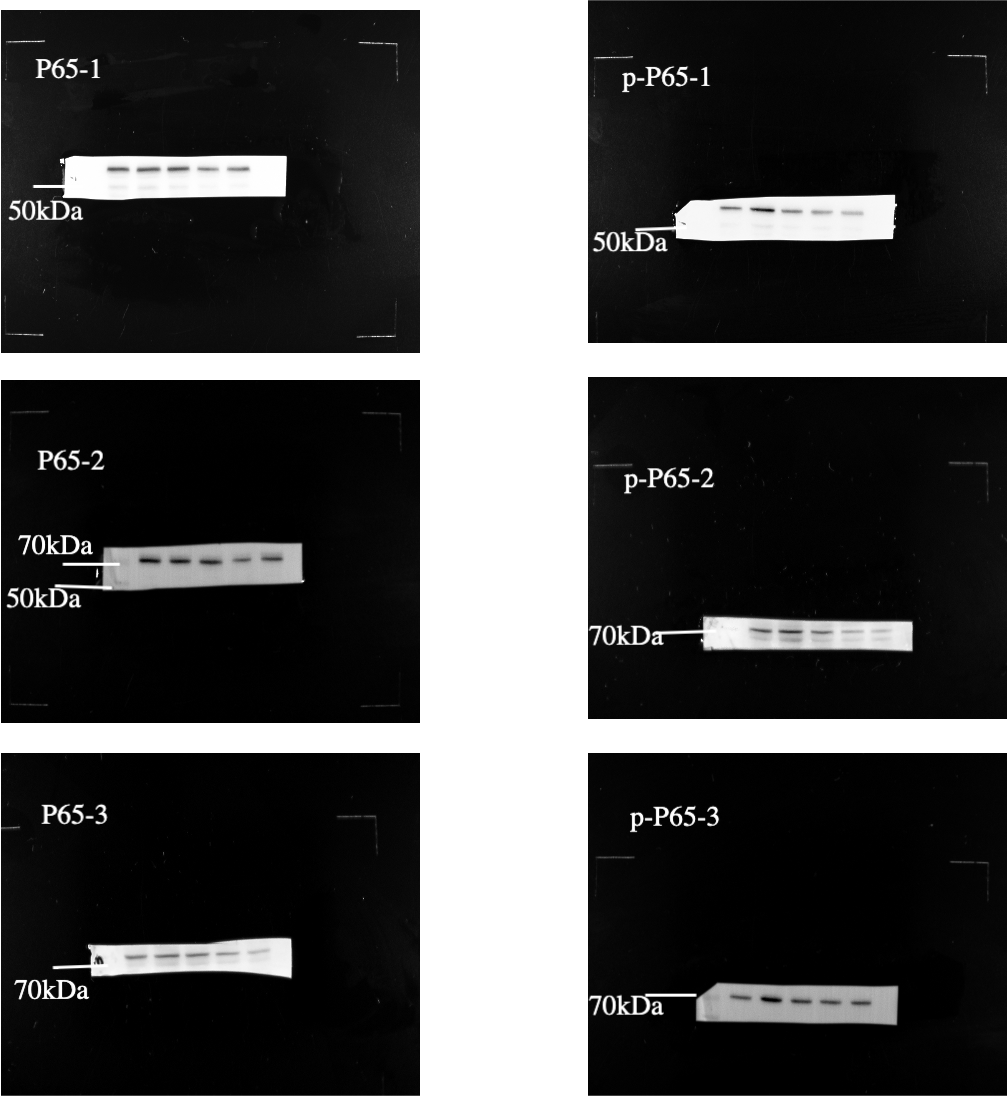


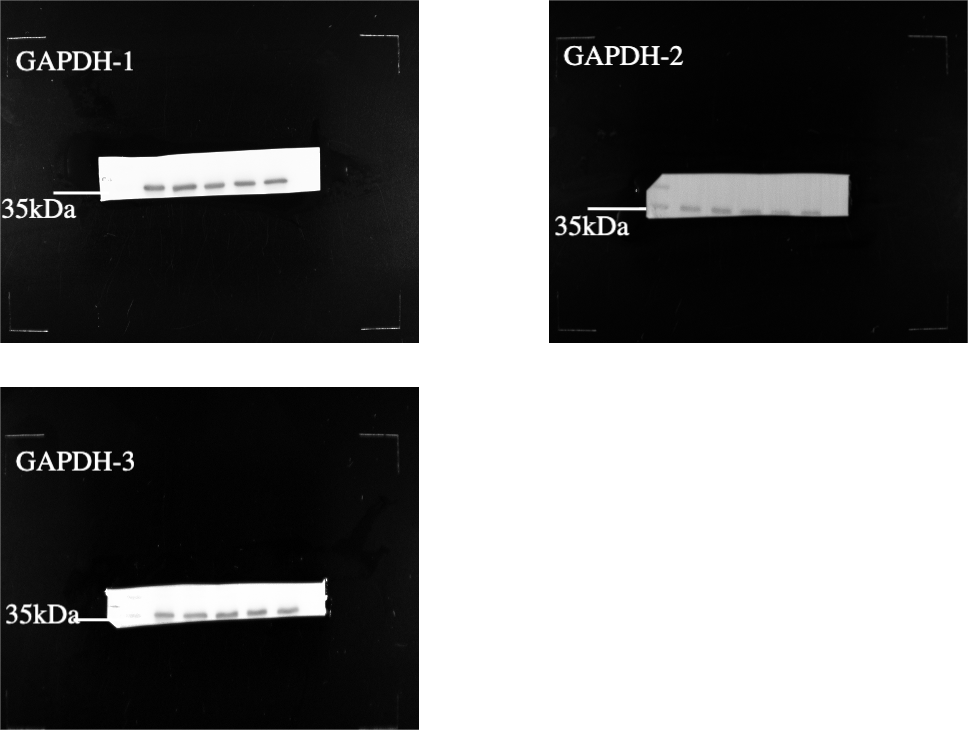


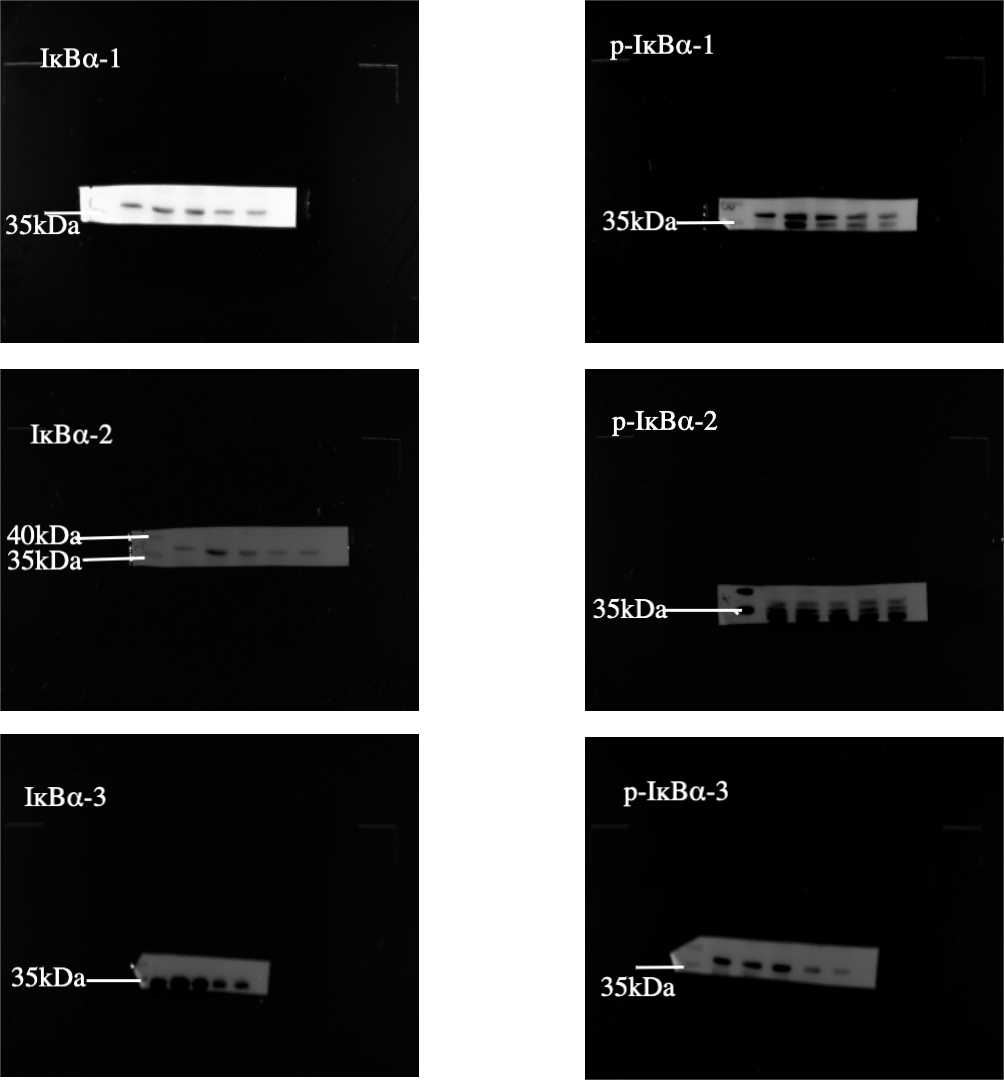


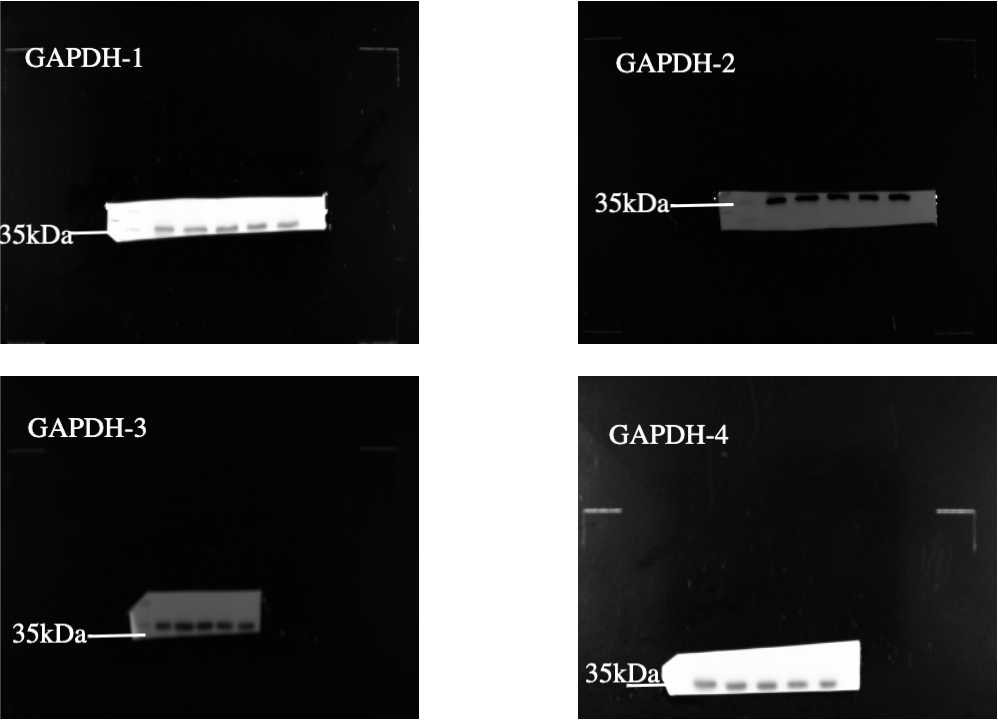


**Figure S22.** The raw western blot images for Figure 6A.
